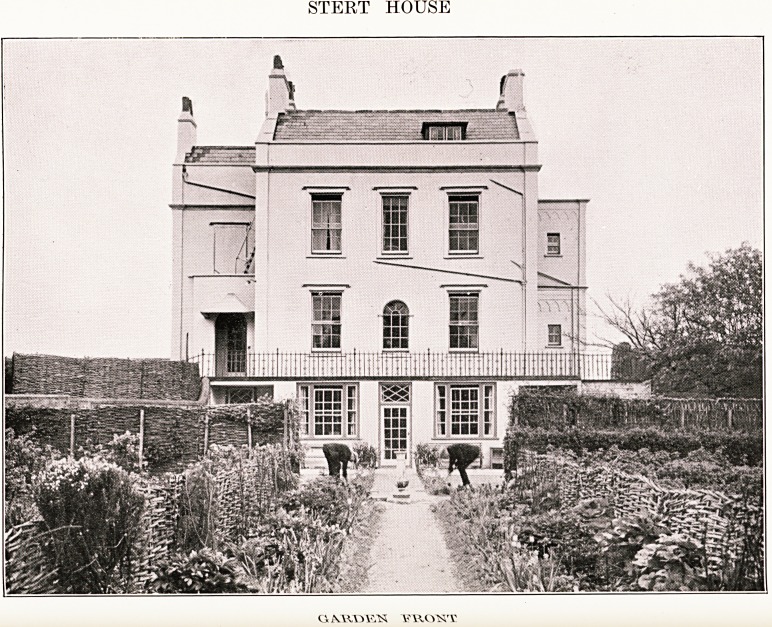# The Medicinal Waters at Burnham-On-Sea

**Published:** 1938

**Authors:** J. A. Nixon, R. H. Brown


### THE MEDICINAL WATERS AT BURNHAM-ON-SEA.

BY

J. A. Nixon, C.M.G., M.D., F.R.C.P.

With a Bibliographical Note by R. H. Brown.

---

Burnham-on-Sea at the present day is chiefly famed as a seaside resort, with splendid sands and sand-dunes, offering admirable schools and (in partnership with Berrow) possessing a championship golf-course. It also gains some reflected glory by reason of Berrow having been the birthplace of this year's "open" golf champion, Mr. Reginald Whitcombe. Nevertheless, Burnham made its first bid for a place in the sunshine about one hundred years ago by claiming to have medicinal waters and by the foundation of a spa.

The Bristol Medical Library has recently purchased a small book, which does not figure in the Surgeon-General's *Index Catalogue* of the Army Medical Library, Washington, nor in those of the Royal Colleges of Physicians or Surgeons in London, nor of the Royal Society of Medicine.

The work is entitled *An Historical Account of the Medicinal Waters or Mineral Springs of Daviesville, at Burnham, near Bridgwater*, by George Henning, M.D., published at Bridgwater by George Awbrey, Fore Street, 1836. The author was one of the physicians of the Taunton and Somerset County Hospital. He published another book, *The Critical Inquiry into the Pathology of Scrofula,* which is possessed by the libraries above-mentioned, and other works designated "etc., etc." on the title page which I have not been able to identify.

The book is a small octavo volume of 115 pages (+xii), bound in blue leather, with the title on the back *Burnham Spa.* The quality of the printing is exceptionally good for the time. A clear and well-cut type face shows to good advantage with careful spacing and generous leading between the lines. The impression is very even, and with a few exceptions, the colour excellent and uniform. Many a product of modern letterpress machinery compares badly from a technical point of view with this example of the work of a small provincial printer of hand-press days. It has been ascertained that George Awbrey was also, at the date of this book, secretary to the Baptist Church at Bridgwater, and it is known that four or five printers have employed his premises in Fore Street since his days, the present occupants being Messrs. W. H. Smith & Son, with a small printing works behind their stationery shop.

The first chapter describes the situation and amenities of Burnham. The Preface opens with the assumption that the Daviesville Spas were already well known.

"The Daviesville Spas, having already acquired great celebrity in the district where they were discovered, it is thought proper to make them more generally known." Of the composition of the waters it is stated:

"Chalybeate waters, which give momentum to the pulse, elasticity to the muscular tissues, appetite to the stomach, strength to the digestion and vigour to the whole animal fabric are well entitled to the term of tonics or strengtheners. This property, being possessed by one of the spas at Daviesville in combination with laxative and other ingredients, admits of its being designated both tonic and aperient."

In this respect the writer would class the water as saline, pointing out that of other saline spas, Cheltenham alone shows similar chalybeate impregnation.

The second of the Daviesville Spas contains "some of the characteristics of a sulphurous impregnation" which would admit of its "being classed with the waters of Harrogate, and Moffat, of Aix-la-Chapelle and Enghien." By artificially "raising the temperature of the sulphureous spa at Daviesville to a parity with that of Barège or Aix," and by increasing its sulphur content, "it undoubtedly may be made equal to those very celebrated springs in point of medicinal efficiency." [One begins to suspect that the "waters" of Daviesville needed a good deal of "fortifying."] "In other complaints where the psychrolusia alone is required, it may be had in apartments, in all respects convenient and comfortable, and either in the form of the shower, or the plunging bath, and of spring water, or that of the sea, warm or cold."

"Psychrolusia" seemed an unfamiliar term in hydrotherapy, but it figures in the latest edition of Dorland's *Medical Dictionary*—"psychrolusia, bathing in cold water: *psychros*, cold; *louein*, to wash." The analysis of the water reveals that it was turbid and slightly yellow; "the smell was very offensive, resembling that of a cesspool, mixed with an odour not unlike bad horseradish." After this frank statement it seems almost irrelevant to mention that it contained muriate of soda, sulphates of magnesia and soda, carbonate of lime, oxide of iron, silex, and vegetable matter. It was apparently very slightly effervescent.

This healing water, which, as the author says, "seemed to possess rather a putrid flavour," was not found flowing from a natural spring. The Reverend David Davies, vicar or stipendiary curate of Burnham, recognizing many points of strong resemblance between the earth of Daviesville and that of Cheltenham, sank a well which at a depth of seventy-five feet "arrived at the spring, which proved to be a mineral water, possessing active medicinal properties, but fortunately contained in a vehicle not repugnant to the most fastidious palate." Truly, our grandparents had strong palates. However, the vicar sank another well a short distance from the first and struck the same water at a depth of only twenty-five feet.

Thus provided with a medicinal water that was not repugnant to the most fastidious palate, Mr. Davies proceeded to develop a spa, to which he gave the name "Daviesville."

"On the western side of the Churchyard and intervenient to it and the Beach is the Esplanade or Terrace, which fronts the hamlet of Daviesville. It extends itself along the coast northward and is computed to be two hundred yards in length and thirty in breadth. . . . Mr. Dodd has led the way by building two tasteful Cottages in . . . the cottage style on the Sandhills, in a line with Daviesville and about half-a-mile distant from it northward. They are in the hamlet of Paradise. . . . The terrace of Daviesville is bounded and upheld by a Sea-wall of masonry of great substance and strength. . . . On this terrace stands the original Pharos or Lighthouse: the great feature of the place and the cynosure of the waters. . . . This eminently important though homely structure was erected in the

ESPLANADE FRONT

### STERT HOUSE

GARDEN FRONT

PLATE XVIII

first year of the present century, at the sole cost and risk of the Reverend David Davies."

"It was the erection of this building," says the author, "that raised Burnham from obscurity. It drew strangers to the place and those of quick perception saw intuitively that it was a land of promise."

In the hamlet of Daviesville a Bath House was built, fitted with sea water baths and the hot baths of the sulphurous spring. Facing the Bath House was "the Machinery for raising the sea water and conveying into cisterns for the supply of the cold and warm baths." There was also an unexpected amenity, viz.: "Cisterns or beds for the feeding and fattening of oysters. The oysters being in the first instance brought from Wales" and fattened by the addition of oatmeal or barley meal to the water. "The constant supply of them at the customary prices cannot fail to be a great acquisition to the neighbourhood and in particular to invalids, to whom it is a nutritious article of diet, and a restorative in emaciating diseases. And here I may be permitted to add that it is the intention of the Reverend gentleman . . . to plant oyster beds in suitable parts of the Bay of Bridgwater."

Dr. Henning gives the distance of Burnham from Bath, Bristol, Glastonbury, Taunton and Bridgwater, with some directions for reaching it, and closes Chapter I with the information: "The Hotel is now about to be re-opened with every convenience; and at Whitburn's Livery Stables, a fly and Bath chair are kept for the use of Visitors. In the Pump Room is a moderate selection of books for general reading, with a London newspaper. There is a post daily. For the accommodation of those individuals who may prefer bathing in the sea at high water, suitable machines are kept, with proper attendants."

There are thirteen chapters in this little book but the remaining twelve deal with the composition of water in general and the healing properties of medicinal waters. The diseases which the Daviesville waters will cure are set forth with the assurance customary amongst prospectus-writers; but the author rises above the ruck by including a disquisition on wearing flannel next the skin. His conclusion is truly judicial: "Now this, as it seems to me, is the *cardo rei*, the precise point to be settled; not whether flannel shall not be worn next the skin when existing circumstances, such as a disproportionate determination of blood to the lungs, a defect of the insensible perspiration or a proneness to the attack of catarrh, require it; but whether having been resorted to, it is at all events to be employed during the rest of life? I think not . . . it enfeebles the frame in delicate persons, especially during the summer months, and more particularly at night. I have, therefore, without hesitation advised the discontinuance of it, and I never knew any harm result."

A charming book, full of appreciation of the climate and sand-dunes of Burnham, that has settled all disputes on the merits of flannel next the skin and brought to light the full-sounding term "psychrolusia" for cold bathing!

A small booklet entitled *Burnham, a Royal Domain of King Alfred*, written and illustrated by Margery B. Sanders, A.R.M.S., gives another account of Burnham Spa:—

Few but the old people remember that for a time Burnham became quite famous as a mineral water spa. It owns a sulphur spring, and a saline chalybeate spring, both very valuable. They are now in the kitchen gardens of the Hall, once the residence of Mrs. King, widow of the late Bishop King, and conveyed to her in 1827. These wells were dug and found by the Rev. David Davies. He was a man of science as well as a benefactor of his sea-side village, and used the money he had received for the rights of his light-house to sink these wells. He was sure the soil showed the existence of saline chalybeate, and was rewarded not only by finding that, but a strong flavour of sulphur also. A second well was sunk and another spring of sulphur was soon discovered. Delighted with his find, he had a Bath House erected, and people began to pour from all over the country to this little place, and even drove in carriages and pairs from Wales to drink and bathe in the mineral waters. The Bath House is still standing, now called Steart House, and is a large Georgian building on the esplanade. The wells are between it and the Berrow Road, with big stables, and pretty wooded gardens which were planted for the patients' use. One delightful path, wooded and gay with flowers, was called till quite recently the Green Walk, and would have come out nearly opposite the footpath across the Berrow Road to Brent Knoll. It is now a private garden and the wells themselves are also in private possession.

Doctor Beverley Morris, who was the last doctor to work them, constantly received letters speaking of the curative power of these waters. An old judge who travelled extensively used to send to him for the bottled water, even when abroad, saying that nowhere else could he get anything so health giving.

There is no printer's name or date of publication in Miss Sanders' pamphlet, but one of the illustrations bears in addition to the author's initials the date 1927.

Mr. Chowins, the Surveyor to the Burnham Urban District Council, states that during 1938 there was a suggestion made that the Spa might be revived.

With this in view the water from one of the wells was analysed and condemned on the ground of heavy contamination with *B. Coli.* The Bath House was recently (27th June, 1938) sold by auction under its present name of Stert House. It is situated near the church with two entrances on the esplanade, and a carriage-drive from the Berrow Road. Between Stert House and the church, on the esplanade, in a short row of houses, stands a small house called "The Round Tower." Part of this house consists of the lower portion of the Pharos or old Lighthouse, which was built by the Rev. David Davies in 1800.

## Figures and Tables

**Figure f1:**
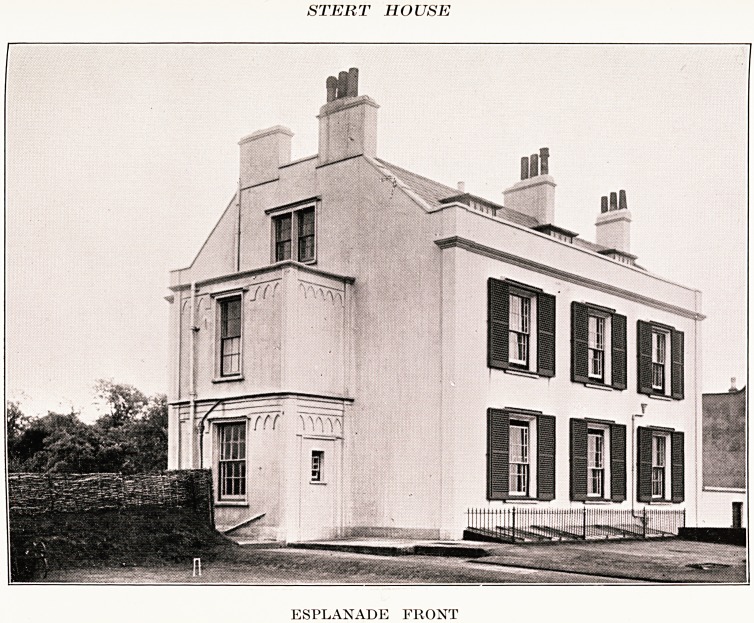


**Figure f2:**